# Metronidazole Interaction with Cu^2+^ and Zn^2+^: Speciation Study in Aqueous Solution and Biological Activity Evaluation

**DOI:** 10.1021/acsomega.4c04166

**Published:** 2024-06-24

**Authors:** Federica Carnamucio, Claudia Foti, Nicola Micale, Natascha Van Pelt, An Matheeussen, Guy Caljon, Ottavia Giuffrè

**Affiliations:** † Department of Pharmaceutics and Center for Pharmaceutical Engineering and Sciences, School of Pharmacy, Virginia Commonwealth University, Richmond, Virginia 23284, United States; ‡ Dipartimento di Scienze Chimiche, Biologiche, Farmaceutiche ed Ambientali, Università di Messina, Viale F. Stagno d’Alcontres 31, 98166 Messina, Italy; § Laboratory of Microbiology, Parasitology and Hygiene (LMPH), Infla-Med Centre of Excellence, University of Antwerp, S7, Universiteitsplein 1, 2610 Wilrijk, Antwerp, Belgium

## Abstract

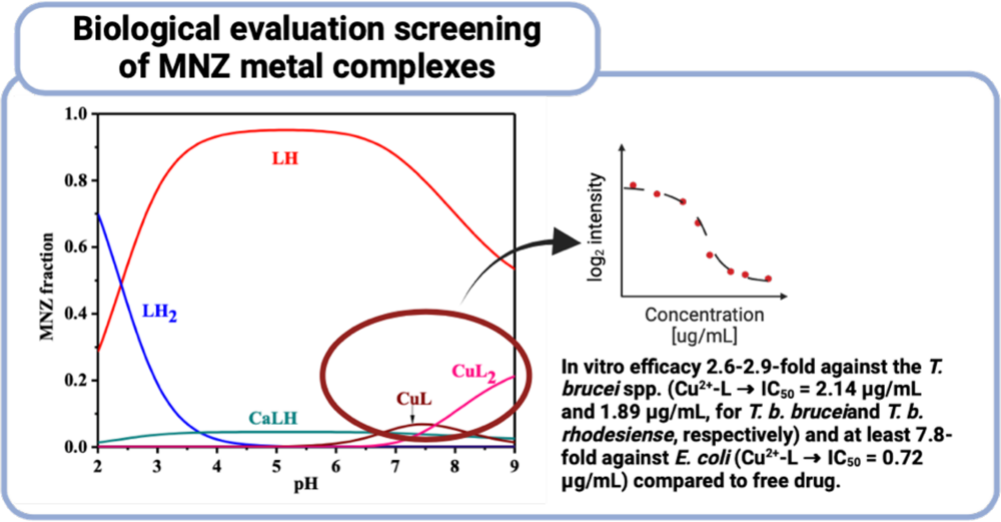

Metronidazole (2-methyl-5-nitro-1*H*-imidazole-1-ethanol, MNZ) is a well-known and widely used drug for its excellent activity against various anaerobic bacteria and protozoa. The purpose of this study is to elucidate the ability of MNZ to form metal complexes with Cu^2+^ and Zn^2+^ and to demonstrate that complexation increases its bioactivity profile against different pathogenic microorganisms. The interaction of MNZ with Cu^2+^ and Zn^2+^ was investigated in NaCl aqueous solution under different conditions of temperature (15, 25, and 37 °C) and ionic strength (0.15, 0.5, and 1 mol L^–1^) by potentiometric and spectrophotometric titrations. The obtained speciation models include two species for the Cu^2+^-containing system, namely, CuL and CuL_2_, and three species for the Zn^2+^-containing system, namely, ZnLH, ZnL, and ZnLOH. The formation constants of the species were calculated and their dependence on temperature and ionic strength evaluated. Comparison of the sequestering ability of MNZ under physiological conditions revealed a capacity toward Cu^2+^ higher than that toward Zn^2+^. A simulation under the same conditions also showed a significant percentage of the Cu^2+^–MNZ species. The biological assessments highlighted that the complexation of MNZ with Cu^2+^ has a relevant impact on the potency of the drug against two *Trypanosoma* spp. (i.e., *T. b. brucei* and *T. b. rhodesiense*) and one gram-(−) bacterial species (i.e., *Escherichia coli*). It is noteworthy that the increased potency upon complexation with Cu^2+^ did not result in cytotoxicity against MRC-5 human fetal lung fibroblasts and primary peritoneal mouse macrophages.

## 1 Introduction

**Figure 1:**
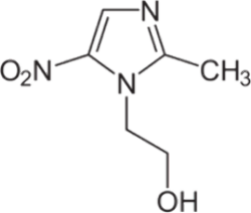
Chemical structure of metronidazole (2-methyl-5-nitro-1*H*-imidazole-1-ethanol, MNZ (L)).

Metronidazole (2-methyl-5-nitro-1*H*-imidazole-1-ethanol, MNZ), shown in Figure 1,^1^ is a 5-nitroimidazole derivative that exhibits great and selective activity against anaerobic bacteria (e.g., *Staphylococcus aureus* and *Escherichia coli*)^2−6^ and some protozoa (e.g., *Entamoeba*, *Trichomonas*, *Trypanosoma*, and *Leishmania* spp.).^7−10^ It has also been shown to have excellent antifungal properties.^11^ Unfortunately, over the years, its overuse has led to the development of MNZ-resistant microorganisms.^12−14^ Among the various methods reported in the literature to overcome the resistance issue,^12^ complexation with transition metals has proven to be one of the strategies with the greatest potential to enhance the bioactivity of MNZ^15^ and protect it from degradation by gastric juice.^16^ The presence of three potential donor atoms (the nitrogen atom of the imidazole ring and the oxygen atoms of the primary alcohol moiety and the nitro group) makes MNZ a good candidate for the coordination with several metal cations such as Mn^2+^, Fe^3+^, Co^2+^, Ni^2+^, Cu^2+^, Zn^2+^, Cd^2+^, Hg^2+^, and Ag^+^.^16,17^ In addition, it is noteworthy that Ru(I)-based MNZ metal complexes have shown good antitumor efficacy in the treatment of lung cancer.^18^ Therefore, the study of the interaction between MNZ and transitional metal cations is interesting to propose new therapeutic approaches. Zn^2+^ and Cu^2+^ are essential trace elements for all living organisms.^19,20^ Both are implicated in numerous biological activities, including modulation of enzymatic and catalytic activities in several oxidation–reduction processes.^16^ The structural role of Zn^2+^ is critical in metalloproteins, which are important in transcription and replication processes and in nucleic acid repair.^21^ Zn^2+^ is also involved in several enzymatic activities necessary for various physiological functions, including protection of sulfhydryl groups from oxidation and inhibition of oxidative processes leading to the formation of reactive oxygen species (ROS).^22^ Cu^2+^ is an essential trace element that functions as a catalytic cofactor in numerous biological processes in both bacteria and humans.^20^ Furthermore, it has recently been pointed out that Cu^+^ and Cu^2+^ have a significant impact on antibiotic activity.^23^ There is growing interest in Cu^2+^ complexes, as it has been shown that they can prevent or even reverse antimicrobial resistance to classical antibiotics.^23−26^ To date, there appear to be few studies on *in vitro* to *in vivo* translation, which is attributed to the lack of knowledge of the molecular mechanisms underlying Cu^2+^–antibiotic interactions under physiological conditions.^23^ So far, the exact mechanism for bacterial damage caused by Cu^2+^ has not been fully explained. Therefore, the aim of this study is to verify the ability of MNZ (or L, which indicates “ligand”) to form complexes with Cu^2+^ and Zn^2+^ ions and to demonstrate that complexation allows for increased drug bioactivity. The study of the interaction between MNZ and both metal cations was carried out by potentiometric and UV-spectroscopic titrations in NaCl aqueous solutions of Cu^2+^– and Zn^2+^–MNZ complexes under different temperatures, ionic strengths, and metal/MNZ molar ratio conditions. Measurements performed at different background salt concentrations (NaCl) and temperature values allowed the study of the dependence of stability constants on ionic strength and temperature. The sequestering ability of MNZ toward Zn^2+^ and Cu^2+^ was calculated under physiological conditions by using the pL_0.5_ parameter, the cologarithm of the ligand concentration that sequesters 50% of the metal cation in trace. Moreover, a simulation under real conditions of plasma pH, temperature, equilibria, and concentrations of the main inorganic constituents is also reported. In order to test the efficacy of Zn^2+^–MNZ and Cu^2+^–MNZ species, a standard *in vitro* screening was carried out against a wide panel of pathogenic microorganisms, including one gram-(−) bacterial strain (*Escherichia coli*), one gram-(+) bacterial strain (*Staphylococcus aureus*), two species of fungi (*Candida albicans* and *Aspergillus fumigatus*), and various protozoa (*Trypanosoma cruzi*, *Trypanosoma brucei brucei*, *Trypanosoma brucei rhodesiense*, and *Leishmania infantum*). In addition, *in vitro* cytotoxicity was evaluated on human fetal lung fibroblasts (MCR-5) and primary peritoneal mouse macrophages (PMM).

## 2 Materials and Methods

### 2.1 Materials

The metal cation solutions were obtained by weighing and dissolving the corresponding salt, zinc(II) chloride monohydrate (Merk, puriss.), and copper(II) sulfate pentahydrate (Fluka, puriss.). All metal solutions were standardized by titration with EDTA (ethylenediamine tetraacetic acid disodium salt, Sigma-Aldrich, purity ≥ 99%). Metronidazole was purchased from Alfa-Aesar/Thermo Fisher (purity ≥ 97%). Solutions of hydrochloric acid and sodium hydroxide used for titrations were prepared by dilution of Fluka ampules and standardized with sodium carbonate (purity ≥ 99.5%, Sigma-Aldrich) and potassium biphthalate (purity ≥ 99.5%, Sigma-Aldrich), respectively. Solutions of sodium chloride were obtained by weighing the salt (Sigma-Aldrich, puriss.), previously dried in an oven at 110 °C for at least 2 h. Distilled water (conductivity <0.1 μS cm^–1^) and grade A glassware were employed for the preparation of all solutions.

### 2.2 Potentiometric Apparatus and Procedure

The potentiometric titrations were performed using a Metrohm model 809 Titrando potentiometer combined with a glass electrode. Each potentiometric system was connected to a PC and the experimental titration data were acquired by the Metrohm TIAMO 2.2 software, which allows control of emf stability, titrant delivery, and data acquisition. The estimated accuracy of the apparatus is ±0.15 and ±0.002 mL for emf and for readings of titrant volume, respectively. Each titration consists of additions of volumes of NaOH standard to 25 mL of the solution containing Cu^2+^ or Zn^2+^, MNZ, HCl, and the supporting electrolyte NaCl. The experimental conditions of the titrations are reported in Table 1. Glass jacket thermostated cells were employed for the measurements performed under different conditions of temperature (15 ≤ *t*/°C ≤ 37) by bubbling pure N_2_ in order to avoid CO_2_ and O_2_ inside the solutions. For each measurement, an independent titration of HCl with standard NaOH was performed to calculate the standard electrode potential *E*^0^ and the p*K*_w_ value under the same experimental ionic strength and temperature conditions.

**Table 1:** Experimental Conditions for Potentiometric and Spectrophotometric Titrations

technique	*t* (°C)	*I* (mol L^–1^)	*C*_M_ (mmol L^–1^)	*C*_L_ (mmol L^–1^)	M/L ratio	pH range
potentiometry	15, 25, 37	0.15–1	1–2	1–4	0.33–1	2–10
UV spectrophotometry	25	0.15	0.04–0.1	0.05–0.08	0.33–1	2–10

### 2.3 UV–Vis Apparatus

The spectrophotometric titrations were carried out with a Varian Cary 50 UV–vis spectrophotometer equipped with an optical fiber with a fixed 1 cm path length. The instrument is interfaced to a PC by Varian Cary WinUV software. By using a Metrohm glass electrode and a Metrohm-Titrando 809 potentiometer, the couple data of absorbance (Abs) and pH vs volume of titrant (mL) were recorded simultaneously for each titration point. All titrations were performed using a thermostated cell to keep the desired temperature under N_2_ to remove CO_2_ and O_2_ from the solutions. The experimental conditions of the titrations are reported in Table 1. For each system, at least four measurements were performed in different conditions of the metal–MNZ ratio. The UV spectra were obtained by scanning from 225 to 400 nm, and a baseline containing only HCl, NaCl, and H_2_O was recorded to consider the contribution of the matrix.

### 2.4 Bacteria and Protozoa Cell Cultures

Briefly, *S. aureus* and *E. coli* were cultured in MHT (Mueller Hinton Broth) and maintained on TSA (Tryptone Soy Agar). *T. cruzi*, Tulahuen CL2 galactosidase strain (nifurtimox- sensitive), was maintained on MRC-5_SV2_ (human lung fibroblast) cells in MEM medium, supplemented with 200 mmol L^–1^l-glutamine, 16.5 mmol L^–1^ NaHCO_3_, and 5% inactivated fetal calf serum. The *T. b. brucei* Squib 427 strain (suramine-sensitive) and *T. b. rhodesiense* STIB-900 strain were maintained in Hirumi’s Modified Iscove’s medium-9 (HMI-9) medium, supplemented with 10% inactivated fetal calf serum. MRC-5_SV2_ cells were cultured in MEM + Earle’s salts-medium, supplemented with 2 mmol L^–1^l-glutamine, 16.5 mmol L^–1^ NaHCO_3_, and 5% inactivated fetal calf serum. All cultures were incubated at 37 °C under an atmosphere of 5% CO_2_.

#### 2.4.1 Activity against *S. aureus* and *E. coli*

The assays were performed at 37 °C in sterile 96-well microtiter plates, each well containing 10 μL of aqueous compound together with a bacterial/fungal/MRC-5 inoculum (190 μL; 5 × 10^3^ CFU/mL). The compounds were tested at 64, 16, 4, 1, and 0.25 μ-mol L^–1^. Bacterial/fungal/MRC-5 growth was compared to untreated-control wells (100% growth) and medium-control wells (0% cell growth). After 3 days of incubation, 50 μL of resazurin was added in each well. After 30 min at 37 °C, the fluorescence was measured at λ_ex_ = 550 nm and λ_em_ = 590 nm. The results are expressed as % reduction in cell growth/viability compared to control wells, and IC_50_ values were determined.

#### 2.4.2 Activity against *T. cruzi*

The assay was performed at 37 °C under an atmosphere of 5% CO_2_ in sterile 96-well microtiter plates, each well containing 10 μL of the aqueous compound together with MRC-5 cell/parasite inoculum (190 μL; 4 × 10^3^ cells/well + 4 × 10^4^ parasites/well). The compounds were tested at 64, 16, 4, 1, and 0.25 μmol L^–1^. Parasite growth was compared to that of untreated-infected controls and uninfected controls. After 7 days of incubation, parasite burdens were assessed after adding the substrate CPRG [chlorophenolred β-d-galactopyranoside; 50 μL/well of a solution of CPRG (15.2 mg); and Nonidet (250 μL) in PBS (100 mL)]. The sample was analyzed spectrophotometrically at 540 nm after 4 h. The results were expressed as % reduction in parasite burden compared to control wells, and an IC_50_ was calculated.

#### 2.4.3 Activity against *T. b. brucei* and *T. b. rhodesiense*

The assay was performed at 37 °C under an atmosphere of 5% CO_2_ in sterile 96-well microtiter plates, each well containing the aqueous compound (10 μL) dilution together with the parasite suspension [190 μL; 1.5 × 10^3^ parasites/well (*T. b. brucei*) or 4 × 10^3^ parasites/well (*T. rhodesiense*)]. The compounds were tested at 64, 16, 4, 1, and 0.25 μmol L^–1^. Parasite growth was compared to untreated-infected controls and uninfected controls. After 3 days of incubation, parasite growth was assessed fluorimetrically after addition of resazurin [50 μL; 50 μg/mL in phosphate buffer] to each well. After 6 h (*T. b. rhodesiense*) or 24 h (*T. b. brucei*) at 37 °C, fluorescence was measured (λ_ex_ 550 nm, λ_em_ 590 nm). The results were expressed as % reduction in parasite growth/viability compared to control wells, and an IC_50_ was calculated.

### 2.5 Calculations

Experimental data of potentiometric titrations were processed by using BSTAC4 and STACO4 programs. The most reliable speciation model for each system was obtained taking into account several factors, namely, the statistical parameters standard and mean deviation, the formation percentages of the species, and the simplicity of the model. The LIANA program was used in order to calculate the parameters for the dependence of formation constant values on temperature and ionic strength. More details on software employed in the refinement of the experimental data are reported in ref (27). The HySS program was used to plot the speciation diagrams.^28^

## 3 Results and Discussions

### 3.1 Speciation Model for Zn^2+^–MNZ and Cu^2+^–MNZ Systems

**Figure 2:**
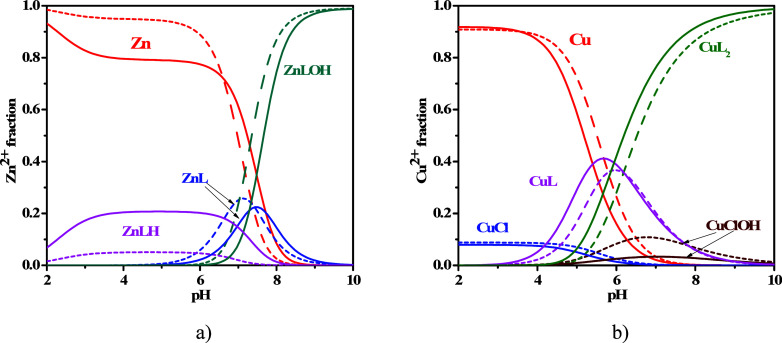
a, b Distribution diagrams at *I* = 0.15 mol L^–1^ in NaCl of (a) Zn^2+^–MNZ(L) and (b) Cu^2+^–MNZ(L) systems at *C*_MNZ_ = 4 mmol L^–1^, *C*_M_ = 2 mmol L^–1^, *t* = 25 °C (solid lines), and *t* = 37 °C (dotted lines).

Both the Cu^2+^–MNZ and Zn^2+^–MNZ systems were investigated via potentiometric titrations. For all calculations, protonation constant values of MNZ(L) and hydrolytic constants of Cu^2+^ and Zn^2+^, as well as Cu–Cl^–^ species formation constant values, were taken into account (see Supporting Information, Tables S1 and S2). Several calculation tests were carried out in order to select the best speciation model for each system, taking into account principles such as model simplicity and improved fit.^29^ The results evidenced that the Cu^2+^–MNZ(L) speciation pattern includes two species, CuL and CuL_2_, and three species for the Zn^2+^–MNZ(L) system, namely, ZnL, ZnLH, and ZnLOH. Formation constant values of Cu^2+^–L and Zn^2+^–L at different temperatures and ionic strength values are reported in Table 2. Unlike the Zn^2+^–L system, the complex species generated by the interaction with Cu^2+^ are characterized by higher stepwise formation constant values, which lead to greater stability of the species and higher formation fractions under the same experimental conditions. More in detail, a comparison between the speciation diagrams under two different temperature values (*t* = 15 and 37 °C) and *I* = 0.15 mol L^–1^ of both systems is shown in Figure 2. In addition, at *t* = 25 °C, the ZnLH species, present in the range of 2 ≤ pH ≤ 8, reaches its maximum formation fraction, corresponding to 0.2, in the range 3 ≤ pH ≤ 7 (see Figure 2a). A notable decrease of its formation fraction is evident at *t* = 37 °C. The two ZnL and ZnLOH species are formed in the ranges 6.5 ≤ pH ≤ 8.5 and 7 ≤ pH ≤ 10, respectively, and reach a maximum of 0.25 at pH = 7.6 (ZnL) and 0.95 at pH = 10 (ZnLOH). Under physiological conditions (*t* = 37 °C, *I* = 0.15 mol L^–1^), a slight increase of formation fraction and a shift of the maximum fraction toward lower pH are distinguishable for both species. As shown in Figure 2b, at *t* = 25 °C, CuL species is formed in the range of 4 ≤ pH ≤ 8 with a maximum of 0.4 at pH = 5.8, while CuL_2_, present in the range of 5 ≤ pH ≤ 10, reaches a maximum of 0.98 at pH = 10. Both species decrease their formation fractions at *t* = 37 °C, also shifting the maximum formation percentage toward slightly higher pH values.

**Table 2:** Experimental Formation Constants of Zn^2+^–MNZ(L) and Cu^2+^–MNZ(L) Species Obtained by Potentiometry

reaction	*t* (°C)	*I* (mol L^–1^)	log β	reaction	log *K*
	15	0.15	13.88(9)^a^		1.88
	25	0.15	13.54(2)		1.87
	25	0.5	14.17(11)		1.66
	25	1	13.96(22)		1.74
	37	0.15	13.02(13)		1.14
	15	0.15	6.36(3)		6.36
	25	0.15	6.57(5)		6.57
	25	0.5	7.40(3)		7.40
	25	1	6.60(8)		6.60
	37	0.15	7.12(2)		7.12
	15	0.15	–0.79(2)		–8.71
	25	0.15	–0.72(2)		–8.44
	25	0.5	0.266(8)		–9.35
	25	1	–0.54(4)		–8.16
	37	0.15	0.09(2)		–8.87
	15	0.15	8.32(10)		8.32
	25	0.15	8.87(4)		8.87
	25	0.5	9.37(4)		9.37
	25	1	8.71(7)		8.71
	37	0.15	8.69(2)		8.69
	15	0.15	16.94(7)		8.62
	25	0.15	17.39(10)		8.52
	25	0.5	18.18(2)		8.02
	25	1	17.65(7)		8.94
	37	0.15	17.12(11)		8.43

a ≥95% of confidence interval.

**Figure 3:**
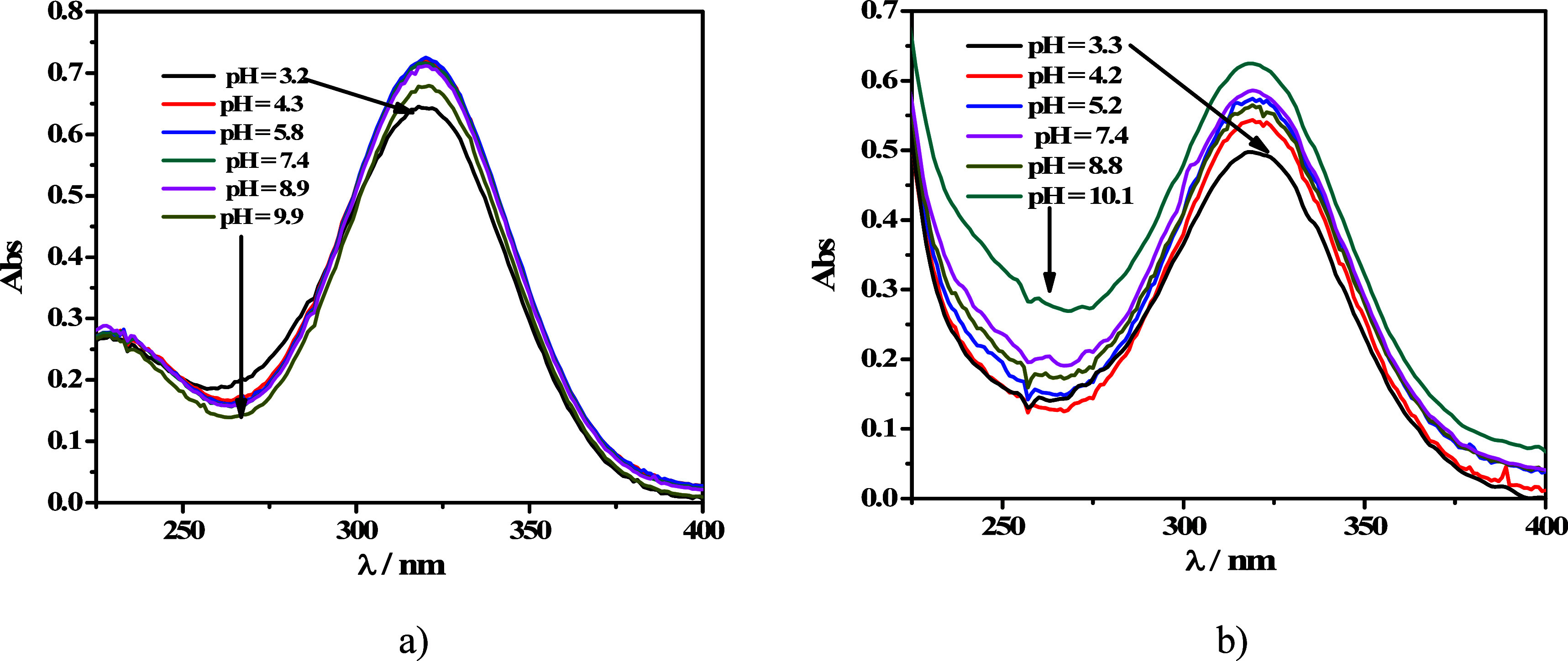
(a, b) Experimental UV spectra at *t* = 25 °C and *I* = 0.15 mol L^–1^ of (a) Zn^2+^–MNZ(L) and (b) Cu^2+^–MNZ(L) solutions at *C*_L_ = 0.05 mmol L^–1^ and *C*_M_ = 0.05 mmol L^–1^.

Both Cu^2+^–L and Zn^2+^–L systems were also analyzed by spectrophotometric titrations to confirm the values of the formation constants and the speciation models. The spectra acquired on the solutions containing Cu^2+^–L and Zn^2+^–L at selected pH in the spectral range 225 ≤ λ ≤ 400 nm are reported in Figure 3a,b. Both systems exhibit a hyperchromic effect as a result of the pH increase. The trend of the spectra referring to solutions containing L and Cu^2+^ or Zn^2+^ is very similar to that of solutions containing only the ligand, as reported in the ref (30). More in detail, an intense UV band with λ_max_ = 319 nm was observed for free MNZ and for the solutions also containing the metal cations. Moreover, the spectra reported in Figure 3a,b clearly indicate that, under the same concentration conditions, the absorbance of solutions containing L and Cu^2+^ is lower than those containing L and Zn^2+^, confirming the greater extent of the interaction with Cu^2+^ compared to that with Zn^2+^.

**Figure 4:**
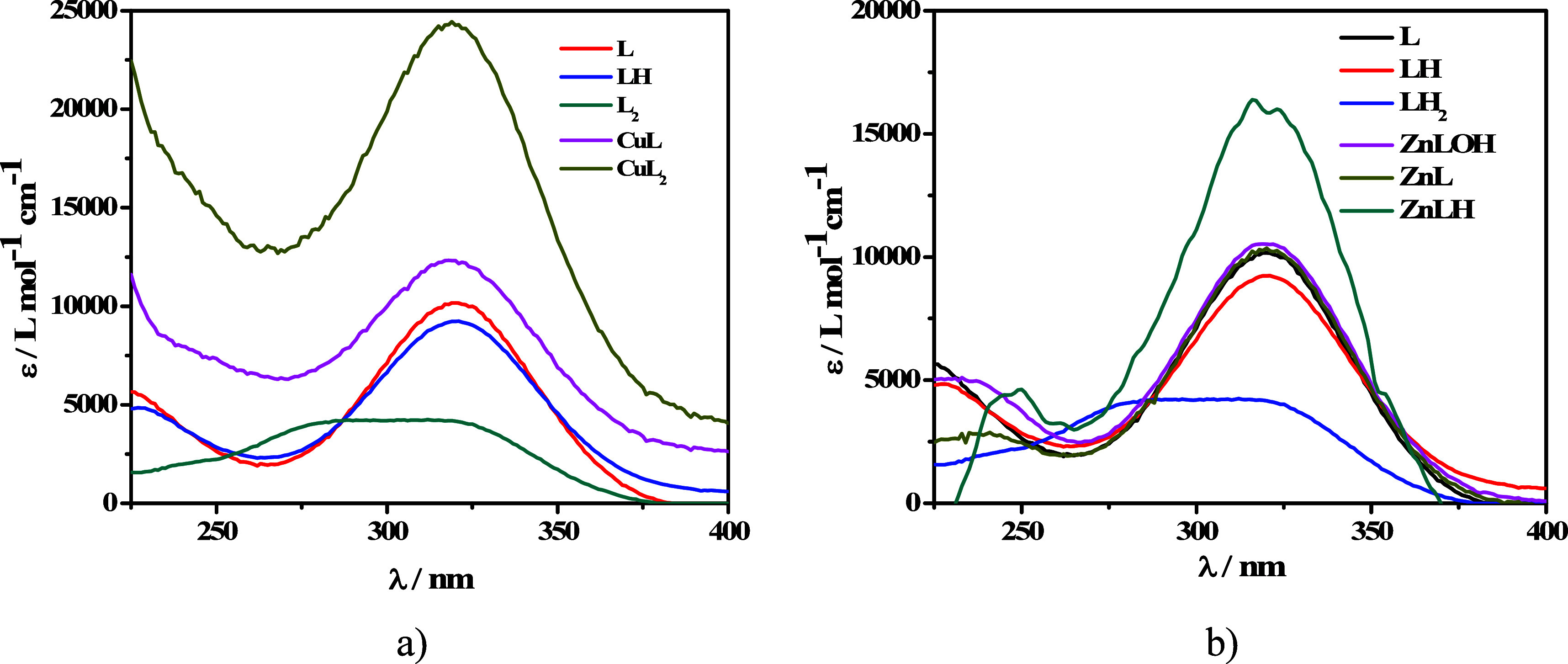
(a, b) ε vs λ of (a) Cu^2+^–MNZ(L) and (b) Zn^2+^–MNZ(L) species at *t* = 25 °C, *I* = 0.15 mol L^–1^.

The trend of the molar extinction coefficients vs λ is shown in Figure 4a,b. CuL and CuL_2_ species reach their maximum peak corresponding to 12500 L mol^–1^ cm^–1^ at λ = 320 nm and 24000 L mol^–1^ cm^–1^ at λ = 320 nm, respectively.

For data processing, the absorption of the main Cu^2+^ hydrolytic species (CuOH^+^) was taken into account, with an ε trend reported in Figure S1 of the Supporting Information. ZnL, ZnLH, and ZnLOH species showed a maximum peak at λ = 325 nm, corresponding to 18500 L mol^–1^ cm^–1^ for ZnLH species, and 10000 L mol^–1^ cm^–1^ for both ZnLOH and ZnL species. The processing of the spectrophotometric experimental data allowed confirmation of both speciation models resulting from the potentiometry and the formation constants of both species relating to the Cu^2+^–L system and of ZnLOH species. The latter is the only species of the Zn^2+^–L system that reaches significant formation percentages under the low concentration conditions employed in the spectrophotometric titrations. Therefore, the formation constants of the other two species, MLH and ML, have been considered in the calculations, and the values obtained by the potentiometry have been kept constant during the process of spectrophotometric data. A comparison between the formation constants gained by potentiometry and spectrophotometry is reported in Table 3.

**Table 3:** Comparison of Experimental Formation Constants of Zn^2+^–L and Cu^2+^–L Species Obtained by Spectrophotometry and Potentiometry at *t* = 25 °C and *I* = 0.15 mol L^–1^

reaction	log β_potentiometry_	log β_UV_	log β_avg_value_
	8.87(4)^a^	8.53(1)^a^	8.70(3)^a^
	17.39(10)	17.20(6)	17.29(10)
	13.54(2)	13.54	13.54(2)
	6.57(5)	6.57	6.57(3)
	–0.72(2)	–0.90(2)	–0.81(2)

a ≥95% of confidence interval.

### 3.2 Dependence of Formation Constant Values on the Temperature and Ionic Strength

As is well-known, temperature and ionic strength have a significant impact on formation constant values. For this reason, determining constants under different ionic strengths is crucial to simulate real fluid conditions. Starting from the knowledge of the formation constant values at different temperatures, the enthalpy change values were calculated by using the following van’t Hoff equation:^31^
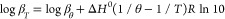
1where log β_*T*_ is the stability constant at a certain temperature (expressed in kelvin), while log β_θ_ is the stability constant at *t* = 298.15 K, and *R* is the universal gas constant expressed as 8.314 J K^–1^ mol^–1^. The values of formation enthalpy changes of all of the species of Cu^2+^–L and Zn^2+^–L systems are collected in Table 4, together with entropy and free energy values. As well-known for electrostatic interactions, it is expected that the entropic contributes to the free energy change should be higher than the one given by enthalpy changes, due to the orientation disorder given by the solvation water molecules. This, considering the parameter values referring to partial reactions, is verified for all the species, except for ZnLH.

**Table 4:** Δ*G*, Δ*H*, and *T*Δ*S* of Cu^2+^–MNZ(L) and Zn^2+^–MNZ(L) Species at *t* = 25 °C and *I* = 0.15 mol L^–1^ in NaCl

reaction	Δ*G*^a^	Δ*H*^a^	*T*Δ*S*^a^
	–49.7	29(9)^b^	79
	–98.7	14(7)	113
	–77.3	–59(11)	18
	–37.5	59(5)	97
	4.6	112(11)	107
	–49.7	29	79
	–49	–15	34
	–77.3	–59(11)	18
	–37.5	59	97
	–47.6	55	103

a In kJ mol^–1^.

b ≥95% of confidence interval.

The dependence of the formation constants on ionic strength was studied by the following Debye-Hückel-type equation:^32,33^
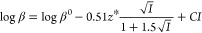
2where β^0^ is the protonation constant at infinite dilution, z* is the parameter that refers to the charges of the species involved in the formation equilibrium, i.e., ∑*z*^2^_reagents_ – ∑*z*^2^_products_, and *C* is an empirical parameter that depends on the stoichiometric coefficients and charges. The formation constants extrapolated to *I* = 0 mol L^–1^, together with the empirical parameter *C* are reported in Table 5.

**Table 5:** Formation Constants at Infinite Dilution and Parameters for the Dependence on Ionic Strength of Cu^2+^–MNZ(L) and Zn^2+^–MNZ(L) Species at *t* = 25 °C in NaCl

reaction	log^*T*^ β	*C*
	9.0(3)^a^	0.1(4)^a^
	17.3(7)	1.3(9)
	15.1(2)	–0.3(2)
	7.3(6)	0.3(8)
	0.01(6)	0.4(8)

a ≥95% of confidence interval.

### 3.3 Sequestering Ability

The tendency of a ligand to complex a free metal cation in solution to form metal–ligand species is expressed as the sequestering ability. To describe the sequestering ability of a ligand toward a certain metal cation, however, the mere knowledge of formation constants is not enough as it is necessary to consider all the possible interactions that both ligand and metal cation may have with all the species present in solution. In order to define it quantitatively, the empirical parameter pL_0.5_ was introduced, which represents the cologarithm of the ligand concentration necessary to sequester 50% of the metal cation in traces.^34,35^ The calculation is based on the following Boltzmann-type sigmoidal equation:
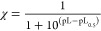
3where χ is the sum of molar fractions of the different complex species and pL is the cologarithm of the total ligand concentration.

**Figure 5:**
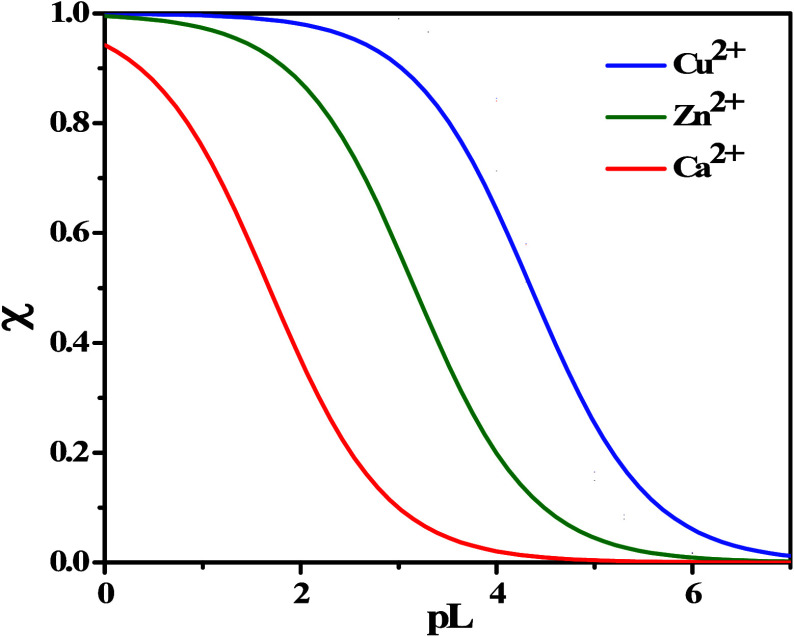
Comparison of sequestering ability of MNZ(L) toward Cu^2+^, Zn^2+^, and Ca^2+^ at *t* = 37 °C, *I* = 0.15 mol L^–1^, and pH = 7.4.

A comparison between the sequestering ability of MNZ toward Zn^2^, Cu^2+^, and Ca^2+^ (one of the main metal cations present in human body and previously studied in our recent work^30^) was evaluated under physiological conditions. According to the calculated pL_0.5_ values at *t* = 37 °C, *I* = 0.15 mol L^–1^, and pH plasma mean value (pH = 7.4), the sequestering ability is significantly higher toward Cu^2+^ (pL_0.5_ = 4.3) than Zn^2+^ (pL_0.5_ = 3.1) and Ca^2+^ (pL_0.5_ = 1.9) (Figure 5).

### 3.4 Simulations under Plasma Conditions

**Figure 6:**
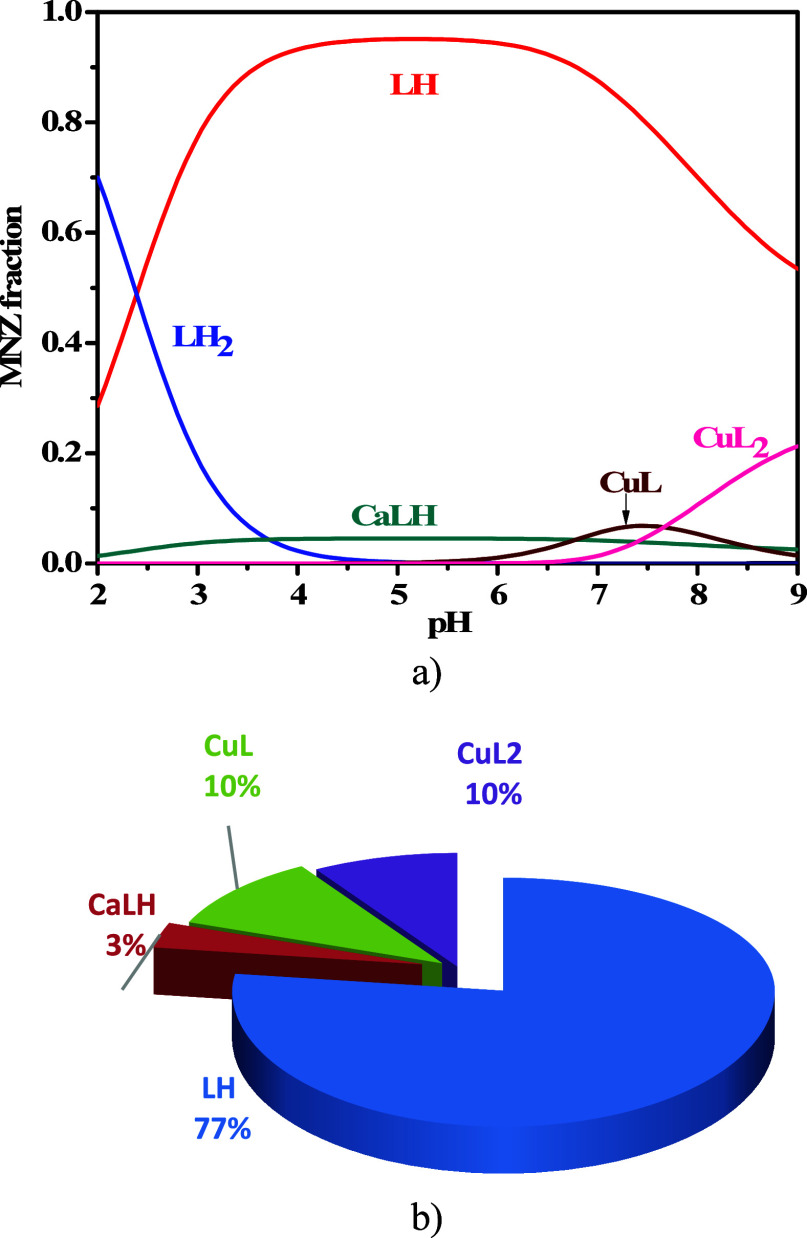
(a, b) Diagrams of MNZ(L) species under plasma conditions (*C*_L_ = 0.06 mmol L^–1^, *C*_Cu_ = 0.017 mmol L^–1^, *C*_Zn_ = 0.02 mmol L^–1^, *C*_Na_ = 140 mmol L^–1^, *C*_K_ = 4.3 mmol L^–1^, *C*_Ca_ = 1 mmol L^–1^, *C*_Mg_ = 1 mmol L^–1^, *C*_Cl_ = 102 mmol L^–1^, *C*_HCO_3__ = 21 mmol L^–1^, *C*_PO_4__ = 0.4 mmol L^–1^, *I* = 0.15 mol L^–1^, *t* = 37 °C): (a) speciation diagram; (b) pie chart at pH = 7.4.

The evaluation of the formation percentages of the complex species under physiological conditions can be an intriguing topic to pursue considering that drug complexation can significantly affect cytotoxicity. MNZ(L) with the most significant electrolytes and their respective equilibria (reported in Supporting Information, Table S3) was considered under plasma conditions (*t* = 37 °C, *I* = 0.15 mol L^–1^, *C*_Zn_ = 0.02 mmol L^–1^, *C*_Cu_ = 0.017 mmol L^–1^, *C*_L_ = 0.06 mmol L^–1^, *C*_Na_ = 140 mmol L^–1^, *C*_K_ = 4.3 mmol L^–1^, *C*_Ca_ = 1 mmol L^–1^, *C*_Mg_ = 1 mmol L^–1^, *C*_Cl^-^_ = 102 mmol L^–1^, *C*_HCO_3_^−^_ = 21 mmol L^–1^, *C*_PO_4__ = 0.4 mmol L^–1^).^36,37^ As shown in Figure 6a,b, both species of the Cu^2+^–L system reach 10% under plasma conditions, while Zn^2+^–MNZ species are not significant.

### 3.5 Antimicrobial and Antiprotozoal Activity

Both Cu^2+^–L and Zn^2+^–L complexes were evaluated for their biological activity profiles against an exhaustive panel of pathogenic microorganisms. Specifically, the antimicrobial activity was assessed against the gram-(−) *E. coli*, the gram-(+) *S. aureus*, the yeast *C. albicans*, and the fungus *A. fumigatus*, whereas the antiprotozoal activity was evaluated against three *Trypanosoma* spp. (i.e., *T. cruzi*, *T. b. brucei*, and *T. b. rhodesiense*) and one *Leishmania* sp. (*L. infantum*). A range of reference compounds (tamoxifen, benznidazole, miltefosine, suramine, doxycycline, flucytosine, and miconazole) were included to demonstrate the validity of the antimicrobial assays (Supporting Information, Table S4). The noncomplexed drug MNZ was used as a main comparator. Salts (chlorides) of Cu^2+^ and Zn^2+^, which can exert their own toxicities, were also used as positive controls. Cytotoxicity assays were performed on both MRC-5 and PMM cells (Table 6). As compared to the pure drug, the complexation with the Zn^2+^ ion did neither affect potency nor selectivity as all IC_50_ values remained above the highest in-test concentration. The interesting outcome of this study came from the complexation of MNZ with the Cu^2+^ ion. In this latter case, the potency of the drug was increased at least 2.6–2.9-fold against the *T. brucei* spp. (Cu^2+^–L → IC_50_ = 2.14 and 1.89 μg/mL, for *T. b. brucei* and *T. b. rhodesiense*, respectively) and at least 7.8-fold against *E. coli* (Cu^2+^–L → IC_50_ = 0.72 μg/mL). More importantly, this increase in drug potency did not affect cytotoxicity against the two healthy reference cell lines. The activity of Cu^2+^–L against other microorganisms remained unaffected as compared to the reference drug (IC_50_ > highest in-test concentration), while showing intriguing pathogen-selectivity probably related to different transport mechanisms within cells.

**Table 6:** *In Vitro* Antiparasitic Activity and Cytotoxicity (IC_50_ and CC_50_ μg/mL, Respectively)

	IC_50_ or CC_50_ (μg/mL)				
	MNZ(L)	Cu^2+^	Zn^2+^	Cu^2+^–L	Zn^2+^–L
*T. cruzi*^a^	>5.64	>2.42	>1.79	>5.33	>6.49
*T. b. brucei*^b^	>5.64	0.75	>1.79	1.89	>6.49
*T. b. rhod*^c^	>5.64	0.94	>1.79	2.14	>6.49
*Sa*^d^	>5.64	>2.42	>1.79	>5.33	>6.49
*Ec*^e^	>5.64	>2.42	>1.79	0.72	>6.49
*L. inf*^f^	>5.64	>2.42	>1.79	>5.33	>6.49
*Ca*^g^	>5.64	>2.42	>1.79	>5.33	>6.49
*Af*^h^	>5.64	>2.42	>1.79	>5.33	>6.49
MRC-5^i^	>5.64	>2.42	>1.79	>5.33	>6.49
PMM^l^	>5.64	>2.42	>1.79	>5.33	>6.49

a *Trypanosoma cruzi* Tulahuen CL2 amastigote stage.

b *Trypanosoma brucei brucei* Squib-427 strain, suramin-sensitive, trypomastigote stage.

c *Trypanosoma brucei rhodesiense* STIB900 strain, drug susceptible, trypomastigote stage.

d *Staphylococcus aureus*.

e *Escherichia coli*.

f *Leishmania infantum* (intracellular amastigotes).

g *Candida albicans*.

h Aspergillus fumigatus.

i Human fetal lung fibroblasts cytotoxicity.

l Primary peritoneal mouse macrophages cytotoxicity.

## 4 Conclusion

An in-depth speciation study of Cu^2+^–L and Zn^2+^–L systems in aqueous solution was carried out in order to point out the thermodynamic behavior of the species and then evaluate their antimicrobial activity. By potentiometric titrations on aqueous solutions, the speciation models and corresponding formation constants of the formed species were determined, which showed the formation of the two species CuL and CuL_2_ and ZnLH, ZnL, and ZnLOH. Both models and formation constants obtained were confirmed using spectrophotometric titrations. With the aim of getting a complete thermodynamic picture, the dependence of the formation constant values on the temperature and ionic strength was also investigated. Moreover, the formation percentages of these species were evaluated by simulating the real plasma conditions of temperature, pH, and ionic strength, and over 50 equilibria between the main electrolytes present in the plasma were considered. According to the biological screening, the L complexation with Cu^2+^ has a significant impact on the potency of the drug against *T. b. brucei*, *T. b. rhodesiense*, and *E. coli*; on the contrary, the presence of Zn^2+^ does not lead to enhanced activity.

## Supporting Information Available

Table S1: Hydrolysis constants of Zn^2+^ at different temperatures and ionic strength values; Table S2: Hydrolysis constants of Cu^2+^ and formation constant values of Cu^2+^-Cl^–^ species at different temperatures and ionic strength values; Figure S1: ε vs λ of CuOH^+^ at *t* = 25 °C, *I* = 0.15 mol L^–1^; Table S3: Formation constant values of the species considered for simulations under plasma conditions; Table S4: List of reference drug and IC_50_ values of the screening panel (PDF)

## Abbreviations

MNZ or L: Metronidazole
*T. cruzi*: *Trypanosoma cruzi*
*T. b. brucei*: *Trypanosoma brucei*
*T. b. rhod*: *Trypanosoma brucei* rhodesiense
*Sa*: *Staphylococcus aureus*
*Ec*: *Escherichia coli*
*L. inf*: Leishmania infantum
*Ca*: *Candida albicans*
*Af*: *Aspergillus fumigatus*
MRC-5: Human fetal lung fibroblasts cytotoxicity
PMM: Primary peritoneal mouse macrophages cytotoxicity
