# Supplementary material for: Metronidazole Interaction with Cu2+ and Zn2+: Speciation Study in Aqueous Solution and Biological Activity Evaluation
Source: ACS Omega. 2024 Jun 24;9(26):29000–8. doi: 10.1021/acsomega.4c04166 (PMC11223215; doi:10.1021/acsomega.4c04166)
Supplement: Supplementary file 1 — ao4c04166_si_001.pdf [file ao4c04166_si_001.pdf]

# Supplementary Information

## Metronidazole Interaction with $Cu^{2+}$ and $Zn^{2+}$: Speciation Atudy in Aqueous Solution and Biological Activity Evaluation

Federica Carnamucio[†*§], Claudia Foti[†], Nicola Micale[†], Natascha Van Pelt[‡], An Matheeussen[‡], Guy Caljon[‡], Ottavia Giuffrè[†*]

[†]*Dipartimento di Scienze Chimiche, Biologiche, Farmaceutiche ed Ambientali, Università di Messina, Viale F. Stagno d'Alcontres 31, 98166 Messina, Italy.*

[§]*Department of Pharmaceutics and Center for Pharmaceutical Engineering and Sciences - School of Pharmacy, Virginia Commonwealth University, Richmond, VA 23284, United States.*

[‡]*Laboratory of Microbiology, Parasitology and Hygiene (LMPH), Infla-Med Centre of Excellence, University of Antwerp, S7, Universiteitsplein 1, 2610 Wilrijk, Antwerp, Belgium.*

**Supplementary Table S1.** Hydrolysis constants of $Zn^{2+}$ at different temperatures and ionic strength values.

| Reaction | $t$ /°C | $I$ / mol $L^{-1}$ | $\log\beta^1$ |
|---|---|---|---|
| $Zn^{2+} + H_2O = ZnOH^+ + H^+$ | 15 | 0.15 | -9.5 |
| | 25 | 0.15 | -9.14 |
| | 25 | 0.5 | -9.15 |
| | 25 | 1 | -9.16 |
| | 37 | 0.15 | -8.78 |
| $Zn^{2+} + 2H_2O = Zn(OH)_2^0 + 2H^+$ | 15 | 0.15 | -17.64 |
| | 25 | 0.15 | -17.10 |
| | 25 | 0.5 | -17.1 |
| | 25 | 1 | -17.22 |
| | 37 | 0.15 | -16.52 |
| $Zn^{2+} + 3H_2O = Zn(OH)_3^- + 3H^+$ | 15 | 0.15 | -29.12 |
| | 25 | 0.15 | -28.4 |
| | 25 | 0.5 | -28.4 |
| | 25 | 1 | -28.47 |
| | 37 | 0.15 | -27.54 |
| $Zn^{2+} + 4H_2O = Zn(OH)_4^{2-} + 4H^+$ | 15 | 0.15 | -41.67 |
| | 25 | 0.15 | -40.40 |
| | 25 | 0.5 | -40.85 |
| | 25 | 1 | -40.38 |
| | 37 | 0.15 | -39.47 |
| $2Zn^{2+} + H_2O = Zn_2(OH)^{3+} + H^+$ | 15 | 0.15 | -9.27 |
| | 25 | 0.15 | -8.70 |
| | 25 | 0.5 | -8.89 |
| | 25 | 1 | -8.89 |
| | 37 | 0.15 | -8.54 |
| $2Zn^{2+} + 6\,H_2O = Zn_2(OH)_6^{2-} + 6H^+$ | 15 | 0.15 | -58.91 |
| | 25 | 0.15 | -57.50 |
| | 25 | 0.5 | -57.53 |
| | 25 | 1 | -57.32 |
| | 37 | 0.15 | -55.9 |

[1] F. Crea, G. Falcone, C. Foti, O. Giuffrè, S. Materazzi. Thermodynamic data for $Pb^{2+}$ and $Zn^{2+}$ sequestration by biologically important S-donor ligands, at different temperatures and ionic strengths, New J. Chem., 2014, 38, 3973.

**Supplementary Table S2.** Hydrolysis constants of $Cu^{2+}$ and formation constants of $Cu^{2+}$-$Cl^-$ complex at different temperatures and ionic strength values.

| **Reaction** | ***t* /°C** | ***I* / mol L$^{-1}$** | **logβ[1]** |
|---|---|---|---|
| $Cu^{2+} + H_2O = CuOH^+ + H^+$ | 15 | 0.15 | -7.9 |
| | 25 | 0.15 | -7.7 |
| | 25 | 0.5 | -7.7 |
| | 25 | 1 | -7.7 |
| | 37 | 0.15 | -7.5 |
| $2Cu^{2+} + H_2O = Cu_2(OH)^{3+} + H^+$ | 15 | 0.15 | -6.41 |
| | 25 | 0.15 | -6.10 |
| | 25 | 0.5 | -6.10 |
| | 25 | 1 | -6.79 |
| | 37 | 0.15 | -5.88 |
| $2Cu^{2+} + 2H_2O = Cu_2(OH)_2^{2+} + 2H^+$ | 15 | 0.15 | -11.13 |
| | 25 | 0.15 | -10.72 |
| | 25 | 0.5 | -10.72 |
| | 25 | 1 | -10.65 |
| | 37 | 0.15 | -10.36 |
| $2Cu^{2+} + 4H_2O = Cu_2(OH)_4^0 + 4H^+$ | 15 | 0.15 | -22.43 |
| | 25 | 0.15 | -21.36 |
| | 25 | 0.5 | -21.36 |
| | 25 | 1 | -21.36 |
| | 37 | 0.15 | -20.7 |
| $Cu^{2+} + Cl^- = CuCl^+$ | 15 | 0.15 | -0.26 |
| | 25 | 0.15 | -0.24 |
| | 25 | 0.5 | -0.23 |
| | 25 | 1 | -0.15 |
| | 37 | 0.15 | -8.54 |
| $Cu^{2+} + 2Cl^- = CuCl_2^0$ | 15 | 0.15 | -0.93 |
| | 25 | 0.15 | -0.9 |
| | 25 | 0.5 | -1.07 |
| | 25 | 1 | -1.0 |
| | 37 | 0.15 | -0.19 |
| $Cu^{2+} + 3Cl^- = CuCl_3^-$ | 15 | 0.15 | -1.95 |
| | 25 | 0.15 | -1.94 |
| | 25 | 0.5 | -2.27 |
| | 25 | 1 | -2.19 |
| | 37 | 0.15 | -0.76 |
| $Cu^{2+} + Cl^- + H_2O = CuClOH^0 + H^+$ | 15 | 0.15 | -6.55 |
| | 25 | 0.15 | -6.28 |
| | 25 | 0.5 | -6.24 |
| | 25 | 1 | -6.34 |
| | 37 | 0.15 | -5.98 |

[1] O. Giuffrè, D. Aiello, D. Chillè, A. Napoli, C. Foti, Binding ability of arsenate towards $Cu^{2+}$ and $Zn^{2+}$: thermodynamic behavior and simulation under natural water conditions, *Environ. Sci.: Processes Impacts*, 2020, 22, 1731.

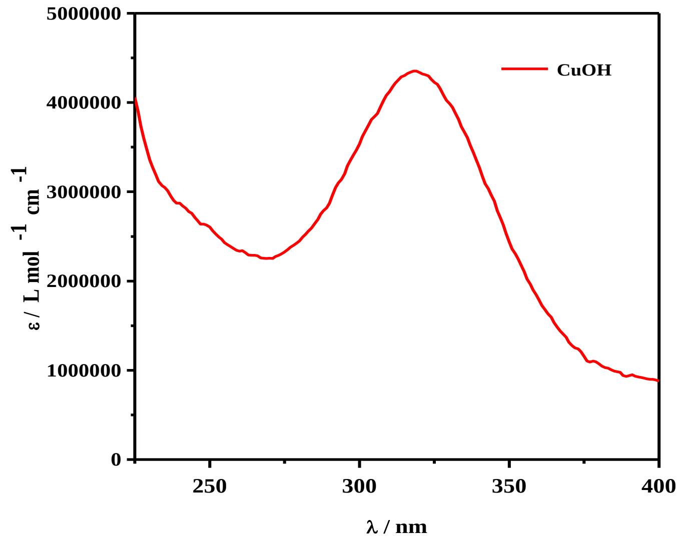


**Supplementary Figure S1.** ε vs. λ of $CuOH^{+}$ at $t$ = 25°C, $I$ = 0.15 mol $L^{-1}$.

**Supplementary Table S3.** Formation constant values of the species considered for simulations under plasma conditions ($t$ = 37°C, $I$ = 0.15 mol $L^{-1}$).

| Reaction | logβ[1] | Reaction | logβ[1] |
|---|---|---|---|
| $Ca^{2+} + H_2O = Ca(OH)^+ + H^+$ | -12.86 | $Cu^{2+} + H_2O = CuOH^+ + H^+$ | -7.5 |
| **MNZ**$^-$ + $H^+$ = MNZH | 11.88[2] | $2Cu^{2+} + H_2O = Cu_2(OH)^{3+} + H^+$ | -5.88 |
| $Ca^{2+}$ + **MNZ**$^-$ + $H^+$ = $CaMNZH^{2+}$ | 13.56[2] | $2Cu^{2+} + 2H_2O = Cu_2(OH)_2^{2+} + 2H^+$ | -10.36 |
| $Zn^{2+}$ + **MNZ**$^-$= $ZnMNZ^+$ | 7.12[2] | $2Cu^{2+} + 4H_2O = Cu_2(OH)_4^0 + 4H^+$ | -20.7 |
| $Zn^{2+}$ + **MNZ**$^-$ + $H_2O$ = $ZnMNZOH^0 + H^+$ | 0.09[2] | $Cu^{2+} + Cl^- = CuCl^+$ | -8.54 |
| $Cu^{2+}$ + **MNZ**$^-$ = $CuMNZ^+$ | 8.69[2] | $Cu^{2+} + 2Cl^- = CuCl_2^0$ | -0.19 |
| $Cu^{2+}$ + 2**MNZ**$^-$ = $CuMNZ_2^{2+}$ | 17.12[2] | $Cu^{2+} + 3Cl^- = CuCl_3^-$ | -0.76 |
| $PO_4^{3-} + H^+ = HPO_4^{2-}$ | 11.64 | $Zn^{2+} + 3H_2O = Zn(OH)_3^- + 3H^+$ | -27.54 |
| $PO_4^{3-} + 2H^+ = H_2PO_4^-$ | 18.47 | $Zn^{2+} + 4H_2O = Zn(OH)_4^{2-} + 4H^+$ | -39.47 |
| $PO_4^{3-} + 3H^+ = H_3PO_4^0$ | 20.50 | $2Zn^{2+} + H_2O = Zn_2(OH)^{3+} + H^+$ | -8.54 |
| $Mg^{2+} + PO_4^{3-} + H^+ = MgHPO_4^0$ | 13.72 | $2Zn^{2+} + 6\ H_2O = Zn_2(OH)_6^{2-} + 6H^+$ | -55.9 |
| $Mg^{2+} + PO_4^{3-} + 2H^+ = MgH_2PO_4^+$ | 19.67 | $Mg^{2+} + CO_3^{2-} = MgCO_3^0$ | 2.22 |
| $Ca^{2+} + PO_4^{3-} + H^+ = CaHPO_4^0$ | 13.58 | $Mg^{2+} + CO_3^{2-} + H^+ = MgHCO_3^+$ | 10.56 |
| $Ca^{2+} + PO_4^{3-} + 2H^+ = CaH_2PO_4^+$ | 19.54 | $Ca^{2+} + CO_3^{2-} = CaCO_3^0$ | 2.56 |
| $Na^+ + PO_4^{3-} = NaPO_4^{2-}$ | 0.95 | $Ca^{2+} + CO_3^{2-} + H^+ = CaHCO_3^+$ | 10.86 |
| $Na^+ + PO_4^{3-} + H^+ = NaHPO_4^-$ | 12.41 | $Na^+ + CO_3^{2-} = NaCO_3^-$ | 0.80 |
| $Na^+ + PO_4^{3-} + 2H^+ = NaH_2PO_4^0$ | 18.69 | $Na^+ + CO_3^{2-} + H^+ = NaHCO_3^0$ | 9.87 |
| $2Na^+ + PO_4^{3-} = Na_2PO_4^-$ | 1.75 | $K^+ + CO_3^{2-} = KCO_3^-$ | 0.61 |
| $2Na^+ + PO_4^{3-} + H^+ = Na_2HPO_4^0$ | 12.13 | $K^+ + CO_3^{2-} + H^+ = KHCO_3^0$ | 9.79 |
| $K^+ + PO_4^{3-} = KPO_4^{2-}$ | 0.85 | $Mg^{2+} + Cl^- = MgCl^+$ | 0.18 |
| $K^+ + PO_4^{3-} + H^+ = KHPO_4^-$ | 12.22 | $Ca^{2+} + Cl^- = CaCl^+$ | 0.03 |
| $K^+ + PO_4^{3-} + 2H^+ = KH_2PO_4^0$ | 18.49 | $Na^+ + Cl^- = NaCl^0$ | -0.5 |
| $2K^+ + PO_4^{3-} = K_2PO_4^-$ | 1.39 | $K^+ + Cl^- = KCl^0$ | -0.48 |
| $2K^+ + PO_4^{3-} + H^+ = K_2HPO_4^0$ | 12.16 | $CO_3^{2-} + H^+ = HCO_3^-$ | 9.85 |
| $Na^+ + K^+ + PO_4^{3-} = NaKPO_4^-$ | 1.93 | $CO_3^{2-} + 2H^+ = H_2CO_3^0$ | 15.97 |
| $Na^+ + K^+ + PO_4^{3-} + H^+ = NaKHPO_4^0$ | 12.45 | | |

[1] Crea, F.; De Stefano, C., Milea, D., Pettignano, A., Sammartano, S, SALMO and S3M: A Saliva Model and a Single Saliva Salt Model for Equilibrium Studies, *Bioinorg. Chem. Appl.* 2015, 2015.
[2] This work.

**Supplementary Table S4.** List of reference drug and $IC_{50}$ values of the screening panel.

| **Screening panel** | | **Reference validation** | |
|---|---|---|---|
| | | **Ref-A** | **$IC_{50}$ (µg/mL)** |
| **1** | MRC-5 | Tamoxifen | 3.72 |
| **2** | *T. cruzi* | Benznidazol | 0.43 |
| **3** | *L. inf* | Miltefosine | 2.05 |
| **4** | *T. b. brucei* | Suramine | 0.03 |
| **5** | *T. b. rhod* | Suramine | 0.07 |
| **7** | *S. aureus* | Doxycycline | 0.08 |
| **8** | *E. coli* | Doxycycline | 0.64 |
| **9** | *C. albicans* | Flucytosine | 0.51 |
| **10** | *A. fumigatus* | Miconazole | 1.18 |
